# The Antifungal Protein AfpB Induces Regulated Cell Death in Its Parental Fungus *Penicillium digitatum*

**DOI:** 10.1128/mSphere.00595-20

**Published:** 2020-08-26

**Authors:** Adrià Bugeda, Sandra Garrigues, Mónica Gandía, Paloma Manzanares, Jose F. Marcos, María Coca

**Affiliations:** a Centre for Research in Agricultural Genomics (CRAG, CSIC-IRTA-UAB-UB), Barcelona, Spain; b Instituto de Agroquímica y Tecnología de Alimentos (IATA, CSIC), Valencia, Spain; University of Georgia

**Keywords:** antifungal proteins, AFP, phytopathogens, *Penicillium digitatum*, regulated cell death, cysteine-rich proteins, cell-penetrating protein, plant protection, fungal infection

## Abstract

Filamentous fungi produce small cysteine-rich proteins with potent, specific antifungal activity, offering the potential to fight fungal infections that severely threaten human health and food safety and security. The genome of the citrus postharvest fungal pathogen *Penicillium digitatum* encodes one of these antifungal proteins, namely AfpB. Biotechnologically produced AfpB inhibited the growth of major pathogenic fungi at minimal concentrations, surprisingly including its parental fungus, and conferred protection to crop plants against fungal infections. This study reports an in-depth characterization of the AfpB mechanism of action, showing that it is a cell-penetrating protein that triggers a regulated cell death program in the target fungus. We prove the importance of AfpB interaction with the fungal cell wall to exert its killing activity, for which protein mannosylation is required. We also show that the potent activity of AfpB correlates with its rapid and efficient uptake by fungal cells through an energy-dependent process. Once internalized, AfpB induces a transcriptional reprogramming signaled by reactive oxygen species that ends in cell death. Our data show that AfpB activates a self-injury program, suggesting that this protein has a biological function in the parental fungus beyond defense against competitors, presumably more related to regulation of the fungal population. Our results demonstrate that this protein is a potent antifungal that acts through various targets to kill fungal cells through a regulated process, making AfpB a promising compound for the development of novel biofungicides with multiple fields of application in crop and postharvest protection, food preservation, and medical therapies.

**IMPORTANCE** Disease-causing fungi pose a serious threat to human health and food safety and security. The limited number of licensed antifungals, together with the emergence of pathogenic fungi with multiple resistance to available antifungals, represents a serious challenge for medicine and agriculture. Therefore, there is an urgent need for new compounds with high fungal specificity and novel antifungal mechanisms. Antifungal proteins in general, and AfpB from *Penicillium digitatum* in particular, are promising molecules for the development of novel antifungals. This study on AfpB’s mode of action demonstrates its potent, specific fungicidal activity through the interaction with multiple targets, presumably reducing the risk of evolving fungal resistance, and through a regulated cell death process, uncovering this protein as an excellent candidate for a novel biofungicide. The in-depth knowledge on AfpB mechanistic function presented in this work is important to guide its possible future clinical and agricultural applications.

## Abstract (precis)

Disease-causing fungi pose a serious threat to human health and food safety and security. The limited number of licensed antifungals, together with the emergence of pathogenic fungi with multiple resistance to available antifungals, represents a serious challenge for medicine and agriculture. Therefore, there is an urgent need for new compounds with high fungal specificity and novel antifungal mechanisms. Antifungal proteins in general, and AfpB from *Penicillium digitatum* in particular, are promising molecules for the development of novel antifungals. This study on AfpB’s mode of action demonstrates its potent, specific fungicidal activity through the interaction with multiple targets, presumably reducing the risk of evolving fungal resistance, and through a regulated cell death process, uncovering this protein as an excellent candidate for a novel biofungicide. The in-depth knowledge on AfpB mechanistic function presented in this work is important to guide its possible future clinical and agricultural applications.

## INTRODUCTION

Fungal infections threaten human health worldwide, causing the death of about 1.5 to 2 million people every year (1). Invasive fungi have fatal consequences in vulnerable patients, such as those immunodepressed by anti-cancer chemotherapies, corticosteroid treatments, HIV/AIDS, or organ transplantation. Fungi also have a significant impact on global food security, damaging more than 20% of crop production, spoiling an estimated 10% of harvested crops, and causing disease in domesticated animals (2). In addition, food safety is challenged by mycotoxigenic fungi that contaminate food and feed with toxins detrimental for health. Consequently, there is a huge demand for fungicides, accounting for a market of $18.7 billion in 2019 that is expected to grow with the emergence of hypervirulent strains and the geographical expansion of pathogens due to global warming and globalization. Only a few classes of fungicides are available today due to the complexity of targeting these eukaryotic microorganisms without affecting plant, animal, or human hosts. These fungicides target the main differences between fungal and host cells, including the plasma membrane and the cell wall composition, ergosterol biosynthesis, or disrupting DNA or RNA synthesis in a fungus-specific manner (3). However, the agricultural application of fungicides has led to the development of resistance to the few available fungicides not only in plant pathogens but also in those causing human diseases (2). The scarceness of licensed fungicides and the emergence of multifungicide resistance make the development of novel antifungal compounds with new modes of action crucial to fight human- and plant-pathogenic fungi.

Filamentous fungi secrete antifungal proteins (AFPs) that specifically inhibit fungal growth without affecting plant or mammalian cell viability (4–10). AFPs are attractive molecules for the development of new fungicides based on their properties (4, 7, 10–12). They are small, cationic proteins that contain six or eight cysteine residues that form three or four disulfide bonds and fold into compact structures, conferring on them high stability and resistance to heat, proteolysis, and extreme pH (5, 13, 14). AFPs are structurally related to defensins, a type of antifungal protein found in insects, plants, and mammalian species, which are integral components of innate immunity (15). AFPs exhibit potent antifungal activity in the micromolar range against important human and plant pathogens (5, 6, 8, 9, 12, 15–18). Furthermore, exploitation of AFPs is feasible, since safe and efficient fungus- or plant-based biofactories have been developed for their production (5, 19, 20).

Detailed knowledge of the mechanistic function of AFPs is an important requisite for their development and application. Notably, variations exist in the spectrum of fungi targeted by different AFPs, demonstrating that they act in a species-specific manner (5, 6, 16, 21). Although AFPs are similar in structure, they differ in amino acid composition and sequence and, presumably, in their mode of action. To date, more than 50 members have been identified in the AFP family, AFP from Aspergillus giganteus and PAF from Penicillium chrysogenum being the first identified and best studied (4, 7, 22). These two proteins are quite similar in structure and antifungal spectrum, but their mechanisms of action are significantly different. Whereas AFP inhibits chitin synthesis and disturbs plasma membrane integrity, PAF is actively internalized in target cells, where it triggers apoptosis (21, 23–25). Divergence in the mode of action makes it necessary to characterize each AFP individually for their efficient biotechnological application as well as for a better understanding of their biological functions.

This work reports a detailed study on the mechanistic function of the AfpB antifungal protein compared to the well-studied PAF. AfpB is a recently identified AFP in the genome of the citrus postharvest pathogen *Penicillium digitatum* (26). The protein could not be detected in natural *P. digitatum* cultures but has been biotechnologically produced in large amounts in the same fungus or in a plant-based system (5, 20). This antifungal protein is very active against major phytopathogenic fungi, especially *Penicillium* species, including the parental *P. digitatum* (5). Moreover, AfpB has been shown to be effective in plant protection treatments against fungal infections, such as *Botrytis cinerea*, the causal agent of gray mold disease in more than 200 vegetable species (6, 20). We show here that AfpB cytotoxicity is determined by interaction with the cell wall of the target fungus and by its active internalization into fungal cells, where it induces a regulated cell death program. This knowledge is crucial for the future development of AfpB as a novel biofungicide.

## RESULTS

### AfpB is a highly active fungicidal protein against its parental fungus *P. digitatum*.

**FIG 1:**
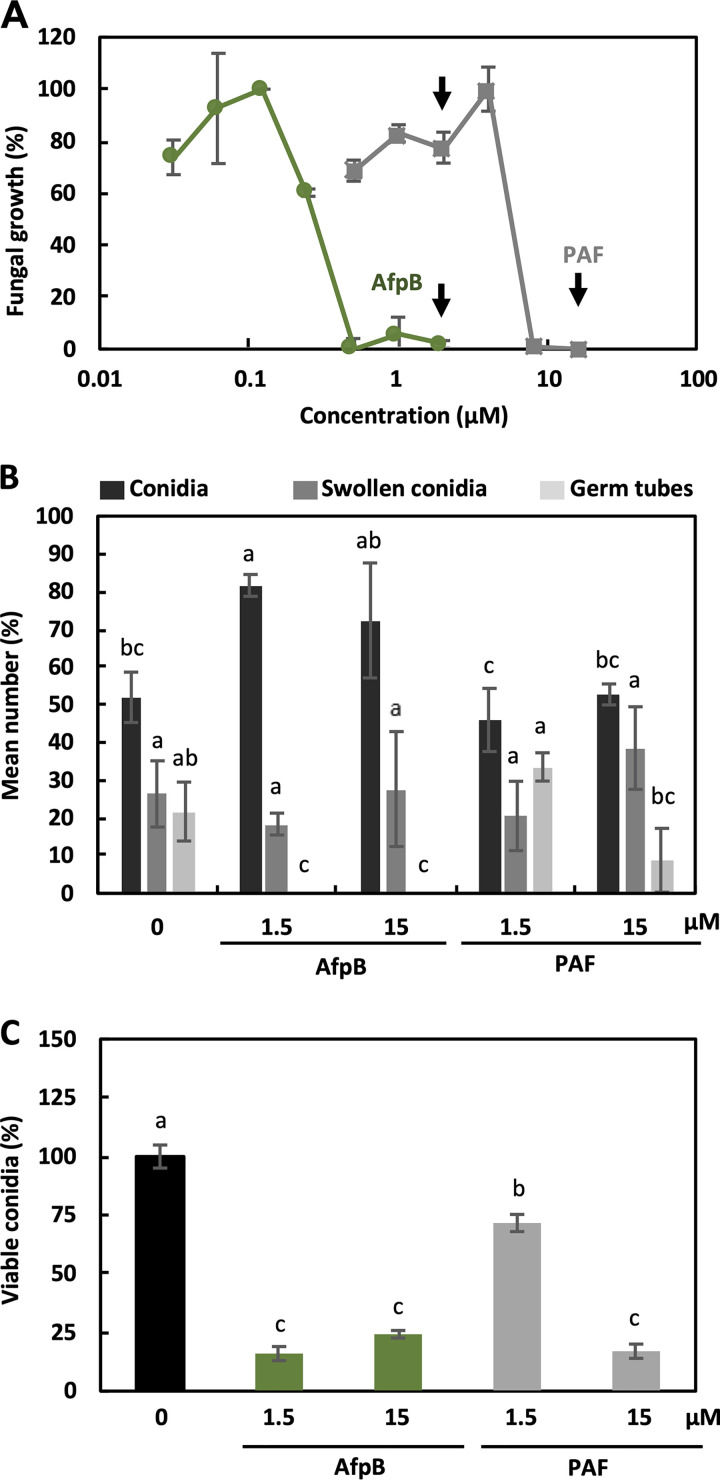
Comparative antifungal activities of AfpB and PAF against *Penicillium digitatum.* (A) Dose-response curves of growth inhibition by AfpB (green) and PAF (gray). Arrows indicate the protein concentrations chosen throughout this work. Curves show the mean percent ± standard deviation (SD) fungal growth of triplicate samples after 72 h of incubation at 25°C. (B) Germination inhibitory activity of AfpB and PAF at the indicated concentrations compared with water. Values are the mean number of conidia, swollen conidia, and germ tubes counted per triplicate after 16 h of treatment. Letters indicate statistical significance (*P < *0.05 by ANOVA and Tukey’s HSD test) with the reference condition in each case. (C) AfpB and PAF have fungicidal activity. Bars represent the percentage of viable *P. digitatum* conidia after 24 h of treatment with AfpB (green) and PAF (gray) at the indicated concentrations compared to the control without any treatment (black). Values are the means ± SD from three independent replicates. Letters show significant differences among the conditions (*P < *0.05 by ANOVA and Tukey’s HSD test).

To better characterize the antifungal protein AfpB, we compared its activity against the parental fungus *P. digitatum* to that of the extensively studied PAF from P. chrysogenum. Both proteins have a similar secondary structure, although there is significant divergence in amino acid sequence, with only around 41% similarity, AfpB being slightly more cationic and hydrophobic than PAF (see Fig. S1 in the supplemental material). They also differ in that PAF is not active against its producer fungus. For comparative purposes, we reevaluated, side-by-side, the growth inhibitory activity of AfpB and PAF against *P. digitatum* (5). AfpB inhibited growth at very low micromolar concentrations, with a MIC of 0.5 μM (Fig. 1A), a level subinhibitory for PAF, which required concentrations more than 16 times higher to fully inhibit growth (Fig. 1A). These results confirmed that AfpB activity is far greater than PAF activity against *P. digitatum.*

10.1128/mSphere.00595-20.1 FIG S1 Amino acid sequence alignment of *P. digitatum* AfpB and P. chrysogenum PAF. Conserved amino acids are shadowed in dark gray and represented with asterisks. Similar amino acids are highlighted in light gray and represented with colons. Cysteine residues are shadowed in black. PAF and predicted AfpB β-sheets are represented as green arrows and labeled β1 to β5. The ɣ-core is boxed. Amino acid alignment was performed using Clustal Omega (https://www.ebi.ac.uk/Tools/msa/clustalo/). The theoretical isoelectric point (pI) and grand average of hydropathicity (GRAVY) were calculated with the Compute pI/Mw and ProtParam tools of the ExPASy Proteomics Server (https://www.expasy.org/). Molecular masses (MM) of AfpB and PAF were experimentally determined previously (5). The number of amino acids (aa) of both proteins is also shown. Download FIG S1, PDF file, 0.4 MB. Copyright © 2020 Bugeda et al. 2020 Bugeda et al. This content is distributed under the terms of the Creative Commons Attribution 4.0 International license.

We also assessed the effect on conidial germination (Fig. 1B). AfpB inhibited *P. digitatum* germination, with no germ tubes detected when conidia were incubated with AfpB at an inhibitory concentration of 1.5 μM, while germlings were clearly visible under control conditions or in the presence of PAF at the same concentration. Inhibitory concentrations of PAF (15 μM) also significantly reduced the number of germ tubes, although some were still visible. These data show that both AfpB and PAF can inhibit conidium germination, although at different concentrations. Because this inhibition could be fungicidal or fungistatic, we compared the effect of these two proteins on conidium viability. As shown in Fig. 1C, AfpB killed the majority of conidia (80%), with a minor proportion of them remaining viable (10 to 20%) and recovering from a 24-h treatment, even at concentrations of 15 μM, 30 times the MIC value. Similarly, at inhibitory concentrations, PAF killed around 80% of conidia, and the remaining conidia recovered when PAF was removed. These results indicate that AfpB as well as PAF growth inhibition can be attributed to both fungicidal and fungistatic activity, with AfpB 1 order of magnitude more active than PAF.

### AfpB is a cell-penetrating peptide at very low concentration.

**FIG 2:**
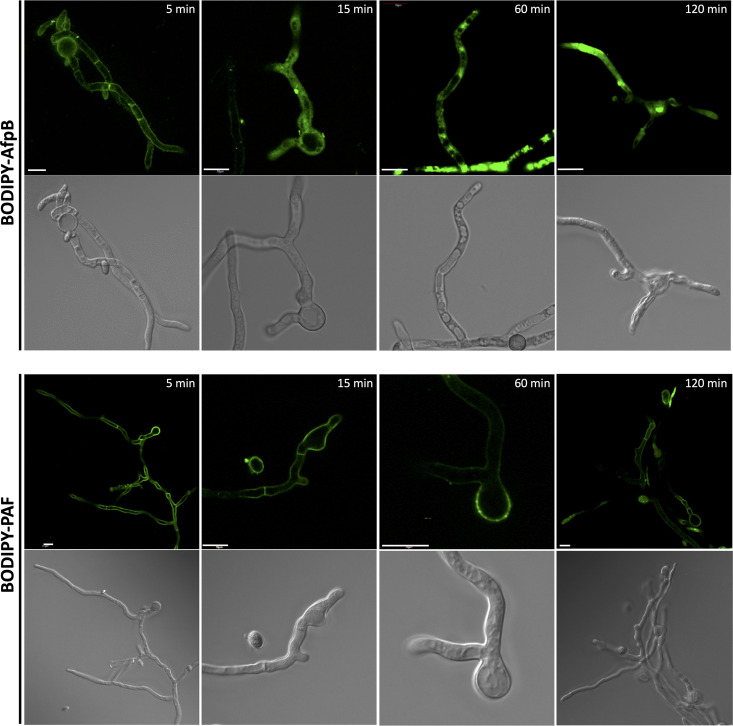
AfpB is a fast cell-penetrating peptide at very low concentrations. Confocal laser microscopy images of the interaction of fluorescently labeled AfpB (BODIPY-AfpB) or PAF (BODIPY-PAF) with *P. digitatum* germlings. Fungal spores were germinated for 16 h and treated with 1.5 μM protein for different periods of time. Upper images correspond to single fluorescent slides and lower images to the bright field images. Bars correspond to 10 μm.

To investigate the mechanism underlying the antifungal activity of AfpB, we compared its interaction with the target fungus to that of the less active PAF protein. For that, we labeled both proteins with the green fluorescent dye BODIPY at their carboxyl groups. After confirming that labeled proteins retained full antifungal activity (Fig. S2), we treated *P. digitatum* germlings with either BODIPY-AfpB or BODIPY-PAF at a protein concentration of 1.5 μM (an inhibitory concentration for AfpB but subinhibitory for PAF) and monitored the interaction by time-lapse live-cell imaging using confocal fluorescence microscopy (Fig. 2). We observed that both proteins localized at the fungal cell surface at early time points (5 to 10 min). AfpB was quickly drawn into the cells, distributed homogenously throughout the cytoplasm and the nucleus, and excluded from vacuoles (15 min onwards), whereas PAF remained longer at the cell surface (up to 120 min). From 60 min onwards, there was high vacuolization and cytosolic BODIPY-AfpB aggregate levels in the hyphae treated with AfpB, indicative of cell deterioration and death. Meanwhile, PAF-treated cells appeared normal, similar to untreated hyphae. It is important to note that both proteins had very similar interaction with the cell wall (detected after 5 min), but there was a delay in PAF entering the cell, which correlates with its lack of inhibition at the subinhibitory concentration.

10.1128/mSphere.00595-20.2 FIG S2 Comparative fungal growth inhibition assays of BODIPY-labeled versus nonlabeled antifungal proteins. Dose-response curves of growth inhibition by AfpB (A) and PAF (B) unlabeled or labeled with BODIPY (BODIPY-AfpB or BODIPY-PAF). Curves show the mean ± SD *P. digitatum* growth, determined by OD_600_ from three independent replicates after 72 h of incubation at 25°C. Download FIG S2, PDF file, 0.2 MB. Copyright © 2020 Bugeda et al. 2020 Bugeda et al. This content is distributed under the terms of the Creative Commons Attribution 4.0 International license.

**FIG 3:**
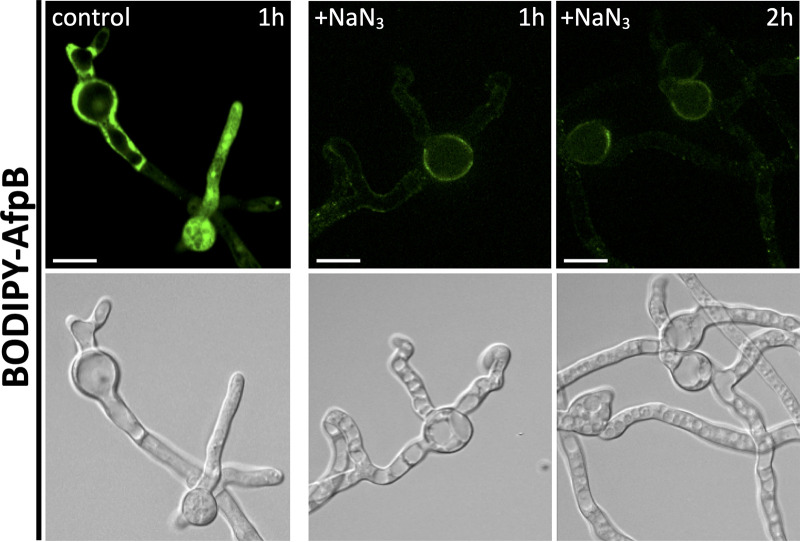
AfpB internalization in *P. digitatum* is energy dependent. Representative images of confocal fluorescence microscopy from fungal germling control or samples treated with NaN_3_ for 15 min and then incubated with BODIPY-AfpB (1.5 μM) for 1 or 2 h. Bars correspond to 10 μm.

The activity of several antifungal peptides has been reported to be energy dependent (16, 27–29). To investigate whether AfpB enters the cells actively or passively, we incubated *P. digitatum* germlings with sodium azide (NaN_3_), an inhibitor of ATPase activity. In the presence of NaN_3_, BODIPY-AfpB remained attached to the cell envelope and could not penetrate the fungal cells (Fig. 3). On the contrary, BODIPY-AfpB rapidly entered the hyphae when not exposed to the inhibitor. Hyphae treated for 2 h with AfpB in the presence of NaN_3_ appeared less damaged than those not exposed to the inhibitor (Fig. 2). These observations suggest that AfpB fungicidal activity is associated with its active entry into the fungal cells.

### Cell wall interaction is important for AfpB antifungal activity and is blocked in a protein mannosylation mutant.

**FIG 4:**
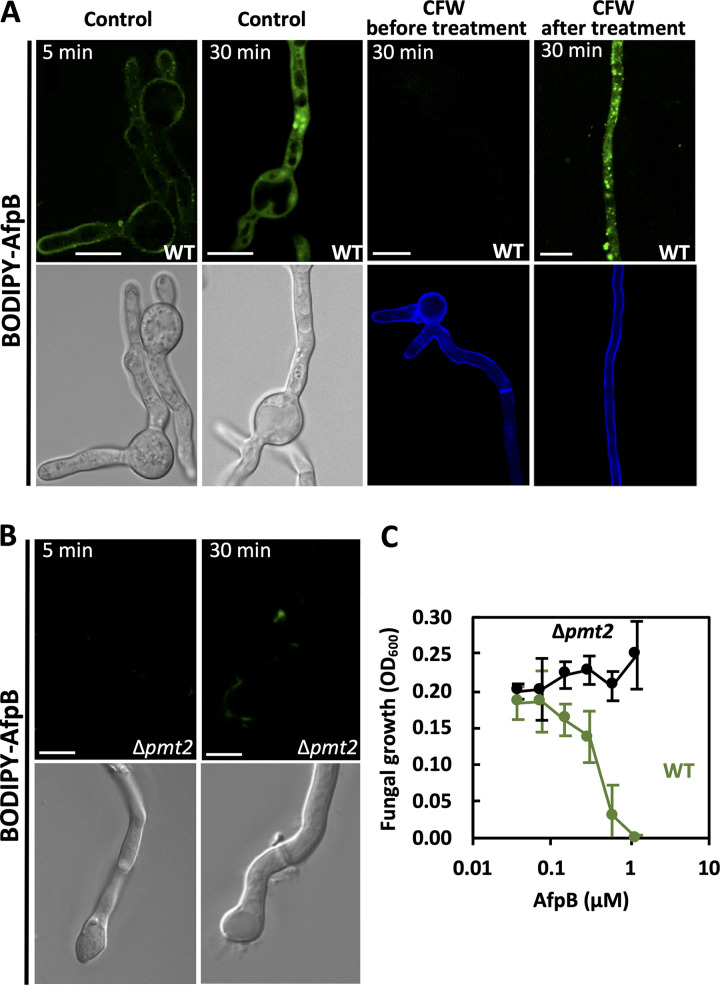
Cell wall interaction is important for AfpB antifungal activity. (A) Representative images of confocal fluorescence microscopy from *P. digitatum* wild-type (WT) germlings, unstained (control) or stained with CFW for 15 min, before or after incubation with BODIPY-AfpB (1.5 μM) for the indicated periods of time. (B) Representative images of the absence of interaction between BODIPY-AfpB (1.5 μM) and *P. digitatum* Δ*pmt2* mutant germlings. (C) Dose response of growth inhibition of *P. digitatum* WT (green) or Δ*pmt2* (black) mutant strain by AfpB. Curves show the means ± SD from triplicate values at an optical density of 600 nm (OD_600_) after 72 h of incubation. Bars correspond to 10 μm.

As a cell-penetrating protein, AfpB must pass through the fungal cell wall to reach the plasma membrane on its way to the cytoplasm. To examine the role of the cell wall in the activity of AfpB, we stained fungal hyphae with calcofluor white (CFW), a fluorescent dye that binds to the chitin polymers of fungal cell walls. CFW prevented AfpB from getting into the intracellular space when hyphae were stained prior to AfpB treatment, whereas CFW staining after treatment did not interfere with AfpB lytic activity (Fig. 4A). These observations indicate that the fungal cell wall has a key role in facilitating AfpB interaction with target cells and, therefore, its activity.

In addition, we characterized the response to AfpB of the *Pdpmt2* deletion mutant strain, which is known to have cell wall defects and greater tolerance to antifungal peptides, such as PAF26 (30). This is a *P. digitatum* null mutant on the O-mannosyltransferase 2 (Δ*pmt2*) that affects protein mannosylation, including the mannoproteins on the outer cell wall. Surprisingly, we did not observe fluorescence on Δ*pmt2* germlings treated with 1.5 μM BODIPY-AfpB, either at the cell wall or intracellularly, at any time point (Fig. 4B). This observation suggests that cell wall mannoproteins are required for the interaction with AfpB. Moreover, growth inhibition assays showed that the Δ*pmt2* mutant was more resistant to AfpB than the wild-type (WT) strain (Fig. 4C). Altogether, these results suggest that mannoproteins determine AfpB interaction with the cell wall and, thus, its antifungal activity.

### AfpB induces rapid ROS production prior to cell death in fungal hyphae.

**FIG 5:**
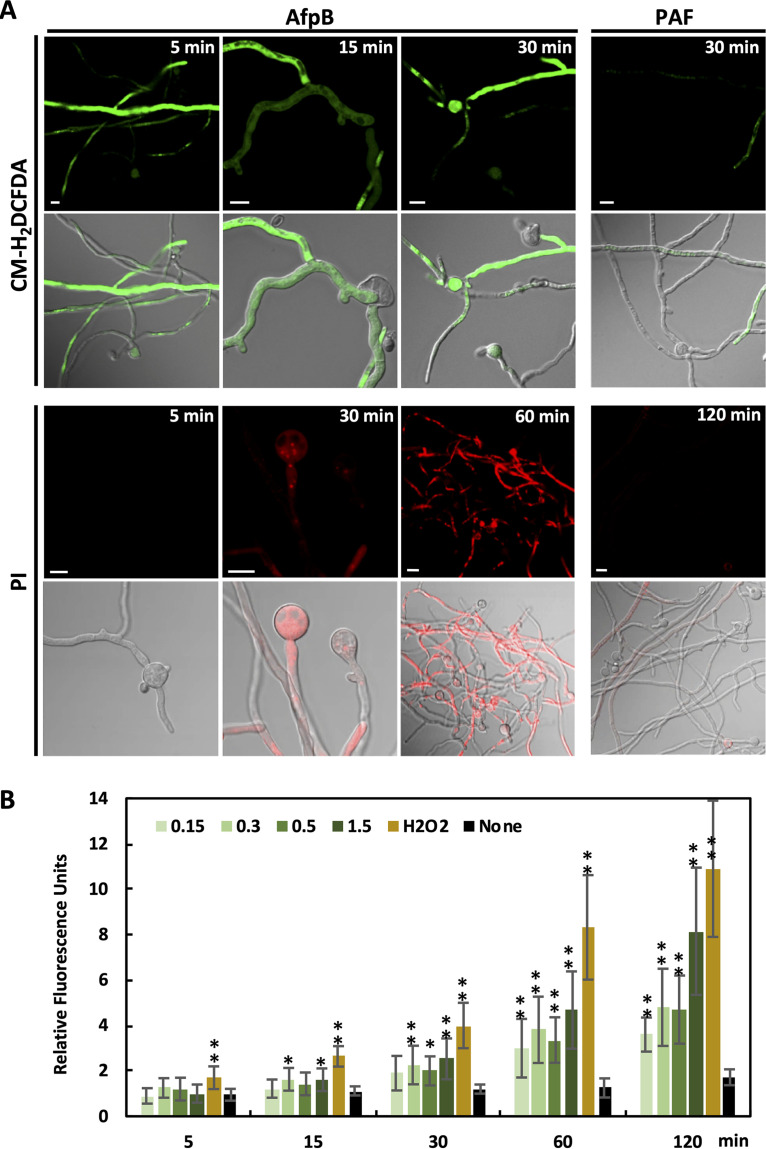
AfpB induces rapid generation of ROS prior to cell death in fungal hyphae. (A) Representative confocal fluorescence images of fungal germlings treated with 1.5 μM AfpB or 1.5 μM PAF and the ROS probe CM-H_2_DCFDA (10 μM) or cell death marker PI (20 μg/ml) for the indicated periods of time. Upper panels are fluorescence images, and lower panels are bright-field merged images. Bars correspond to 10 μm. (B) Quantitative fluorescence of germlings treated with CM-H_2_DCFDA (10 μM) and AfpB at indicated micromolar concentrations (green bars), H_2_O_2_ (5 mM, orange bars), or water (none, black bars) for the indicated time periods (min). Bars are the mean values ± SD from 6 replicates and 2 independent assays (*n* = 12). Asterisks denote statistically significant differences with the control samples (**, *P* < 0.01; *, *P* < 0.05; both by Tukey’s HSD test).

The generation of reactive oxygen species (ROS) is associated with cell death, acting either as signaling molecules in regulated cell death or as toxic compounds causing oxidative damage to lipids, proteins, and nucleic acids and uncontrolled cell destruction. Several studies have previously reported the production of ROS in fungal cells triggered by AFPs (29, 31–34). ROS production was monitored in these studies after long and high-concentration treatments, with deleterious effects observed; it was unclear whether ROS was signaling or a consequence of cell death. To study the oxidative status of hyphae treated with AfpB, we used the permeable fluorescent CM-H_2_CFDA probe as an indicator of ROS cell content (35). Fluorescent hyphae were visible immediately after the addition of AfpB, whereas fluorescence was barely detected in PAF-treated hyphae at the same concentration (1.5 μM) (Fig. 5A). These observations show that AfpB induces the rapid generation of ROS in target fungal cells. However, staining with the cell death marker propidium iodide (PI), which fluoresces upon interaction with nucleic acids and penetrates only cells with damaged plasma membranes, showed red fluorescent hyphae only visible at late times of exposure (Fig. 5A). These permeabilized hyphae could be detected from 30 min, although most frequently they were seen after 1 h of AfpB treatment. Similarly, SYTOX green, which fluoresces upon binding to nucleic acids of hyphae with compromised plasma membrane, was only visible from 30 to 40 min after AfpB treatment (Fig. S3). In contrast, no dead hyphae were observed after 2 h of PAF treatment at the same concentration (Fig. 5A). These results indicate that cell death mediated by AfpB is delayed with respect to ROS production, suggesting that ROS acts on cell death signaling rather than as a consequence of uncontrolled cell death.

10.1128/mSphere.00595-20.3 FIG S3 AfpB-induced membrane permeabilization is delayed with respect to ROS production. Representative images of confocal fluorescence microscopy from *P. digitatum* germlings treated with 1.5 μM AfpB and ROS marker CM-H2DCFDA (10 μM) or SYTOX green (5 μM) for the indicated times. Bars correspond to 10 μm. Download FIG S3, PDF file, 0.5 MB. Copyright © 2020 Bugeda et al. 2020 Bugeda et al. This content is distributed under the terms of the Creative Commons Attribution 4.0 International license.

We quantified the ROS production triggered by AfpB using a fluorimeter (Fig. 5B). ROS accumulation was detected as early as 15 min after AfpB treatment at different concentrations. ROS levels increased progressively over time, reaching values similar to those of hydrogen peroxide (H_2_O_2_) at 5 mM, used as a control. Interestingly, ROS was also detected at low concentrations of AfpB (0.15 μM), concentrations that are not fungal inhibitory, suggesting once again that ROS generation triggered by AfpB has a signaling role instead of a toxic effect.

### AfpB induces regulated death in *P. digitatum* cells.

**FIG 6:**
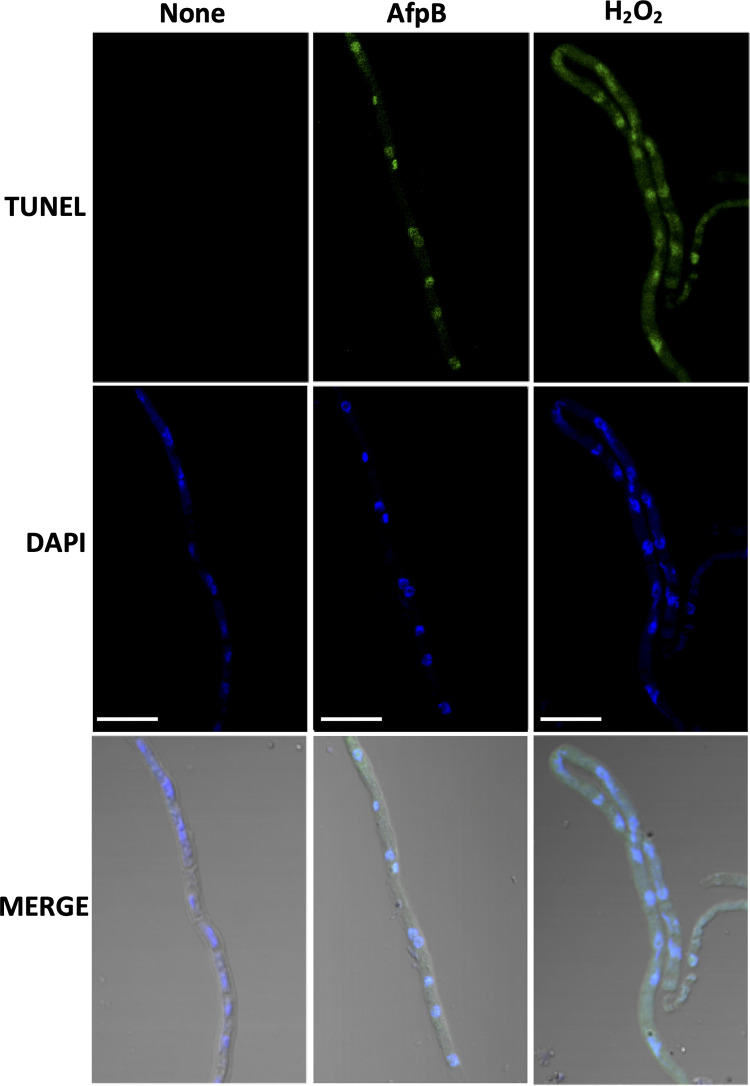
AfpB induces DNA fragmentation in *P. digitatum.* Representative images of confocal fluorescence microscopy from *P. digitatum* germlings without treatment, treated with AfpB (0.5 μM), or treated with H_2_O_2_ (5 mM) for 30 min and stained with TUNEL and DAPI, as indicated. Lower panels correspond to merged images with bright fields. Bars correspond to 10 μm.

The cleavage of linker regions between nucleosomes by nucleases is a hallmark of regulated cell death (RCD). To further investigate the cell death induced by AfpB in *P. digitatum*, we assessed DNA fragmentation in AfpB-treated hyphae compared to hyphae treated with H_2_O_2_, an inducer of the apoptosis phenotype in yeast and filamentous fungi when applied at minimum, but toxic, concentrations (36, 37). The terminal deoxynucleotidyl transferase-mediated dUTP nick-end labeling (TUNEL) assay is one of the most reliable methods for detecting DNA breaks. Fungal hyphae treated with AfpB showed positive TUNEL staining (Fig. 6), with hyphae treated with H_2_O_2_, used as a positive control of DNA fragmentation, giving a similar pattern of staining. The TUNEL-stained regions colocalized with the nuclei visualized by 4′,6-diamidino-2-phenylindole (DAPI) staining. These observations showed that AfpB induces DNA fragmentation in nuclei, which is indicative of RCD.

**FIG 7:**
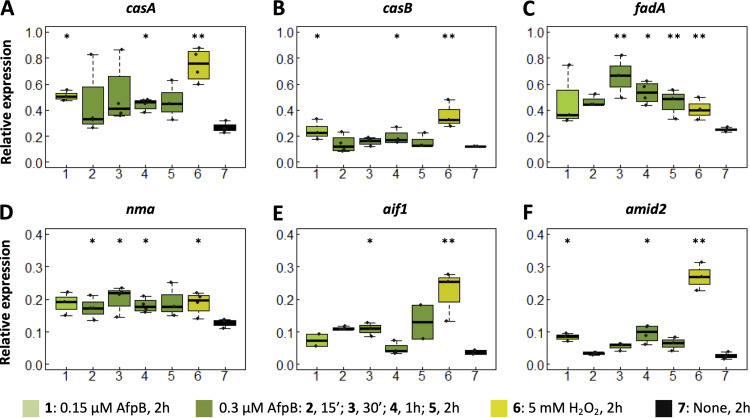
AfpB regulates cell death gene expression in *P. digitatum* hyphae. (A to F) Expression levels of the indicated regulated cell death genes, as determined by qRT-PCR and normalized to the housekeeping β-*tubulin* gene, in *P. digitatum* overnight-grown mycelia, nontreated or treated with AfpB or H_2_O_2_ at the indicated concentrations and time lapses. Graphs show boxplots of at least three values, corresponding to three independent assays with three technical replicates each (*n* = 9). Values from AfpB-treated samples are shown in greenish colors (lanes 1 to 5), from the RCD positive control, corresponding to H_2_O_2_-treated samples, in yellow (lane 6), and from the negative control, corresponding to untreated samples, in black (lane 7). Asterisks denote statistically significant differences compared to untreated samples (*, *P* ≤ 0.05; **, *P ≤ *0.01; both by Student’s *t* test).

Further insights into the intracellular molecular events underlying AfpB-mediated fungal cell death were obtained by monitoring the expression of RCD-associated genes. In these experiments, we used an inhibitory concentration of AfpB (0.3 μM) that was slightly lower than the MIC (0.5 μM), since the AfpB cytotoxic effects were too strong at the MIC, making it difficult to monitor gene expression. Among the genes associated with RCD, caspase-like genes are good candidates, as caspase-like activities are involved in cell death execution. Although fungi do not encode true caspases, fungal metacaspases with different contributions to cell death have been identified (38–40). We identified two metacaspases encoded in the genome of *P. digitatum*, CasA and CasB. Our gene expression analysis revealed that both metacaspases were induced in hyphae in response to AfpB treatment, even at a subinhibitory AfpB concentration (0.15 μM), although at lower levels than those induced by H_2_O_2_ treatment, used as an RCD control (Fig. 7A and B). Similarly, we analyzed the expression of the *fadA* gene encoding the α-subunit of a heterotrimeric G-protein that mediates RCD in response to PAF, *Neosartorya fischeri* NAFP, or the antifungal protein osmotin from plant (23, 41–43). The expression of *fadA* was induced by AfpB treatment, even at higher levels than those caused by H_2_O_2_ treatment (Fig. 7C). Analysis of the *nma* gene, the orthologue of the human *HtrA2* proapoptotic gene, which also induces apoptotic markers in filamentous fungi (44), revealed low but constant induction by AfpB at the different time points at levels similar to those of our positive control (Fig. 7D). The apoptosis-inducing factor gene (*aif1*), as well as the *aif*-homologous mitochondrion-associated inducers of death gene (*amid2*) (45), components of the caspase-independent pathway of RCD in fungi, were also induced in response to AfpB treatment. However, the expression level of the genes was lower than that induced by H_2_O_2_ and was statistically significant only at certain time points (Fig. 7E and F). Altogether, our gene expression results indicate that AfpB regulates cell death in *P. digitatum* through a complex intracellular process, and membrane permeabilization is the consequence, not the cause, of its antifungal activity.

## DISCUSSION

In this study, we demonstrate the strong, rapid antifungal activity of AfpB against *P. digitatum* at concentrations lower than the micromolar level through multiple targets, including components of the cell wall, the plasma membrane, and intracellular elements, which would hinder the development of fungal resistance. Interestingly, we found that the antifungal effect of AfpB occurs inside fungal cells, where it activates a transcriptional reprogramming to trigger cell death. This mechanism of regulated cell killing is less aggressive to hosts than the membranolytic actions of other antifungals, and this, together with its potency and specificity toward fungal cells, makes this protein an excellent candidate to be developed as an antimycotic in clinical therapies as well as a biofungicide for crop and postharvest protection.

**FIG 8:**
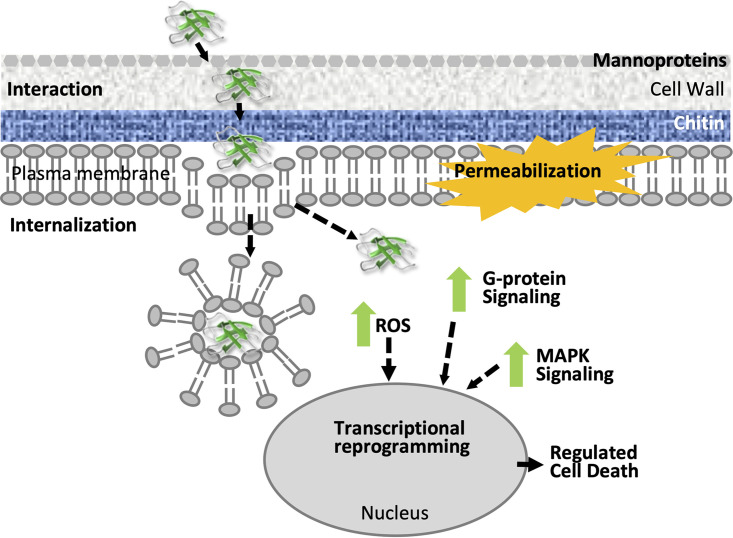
Model of AfpB mode of action. A three-stage process leading to fungal cell death is suggested: (i) interaction with the cell wall, (ii) internalization inside the cell by crossing the plasma membrane, and (iii) intracellular regulated events leading to cell death, including the production of ROS, mitogen-activated protein kinase (MAPK), and G-protein signaling, transcriptional reprogramming, and, eventually, plasma membrane permeabilization.

The side-by-side study of the mode of action of AfpB compared with the well-characterized and less active PAF protein against the phytopathogen *P. digitatum* indicates that both proteins have a pathway of action that fits our previously proposed model for the antifungal action of short synthetic peptides (46, 47). Following this model, AfpB acts through a three-stage process to lead to fungal cell death: first, the interaction with the cell wall, then the energy-dependent cell penetration, and, finally, a series of intracellular regulated actions that end with cell collapse (Fig. 8). This mechanism differs from the one elucidated for AFP from A. giganteus (4, 21, 24) and other antifungal proteins, such as plant defensins (48–51), which act by disrupting plasma membrane integrity, causing cell disintegration and death. However, other AFPs have a cell-penetrating mode of action similar to that of AfpB, including PAF, and P. chrysogenum AfpB orthologs, PAFB, and PgAFP and NAFP from *N. fischeri* (16, 23, 43, 52).

Interestingly, we found that AfpB interaction with the cell wall is important for its activity by facilitating access to the plasma membrane and subsequent entry into the fungal cell (Fig. 8). The fungal cell wall is composed of an outermost highly glycosylated layer of mannoproteins, a thick intermediate layer of β-glucans, and a thin layer of chitin next to the plasma membrane. Staining *P. digitatum* cell walls with CFW, which binds chitin, interfered with the internalization of AfpB, indicating that a fortified cell wall can act as a barrier impeding AfpB diffusion from reaching the fungal plasma membrane, or that AfpB requires chitin interaction, blocked by CFW. Varied susceptibilities to AfpB have been observed in *P. digitatum* chitin synthase mutants (53), which suggests that both alternatives are feasible. Similarly, the A. giganteus AFP binds chitin, and reinforcement of the cell wall confers tolerance to AFP (54). Additionally, we show that the Δ*pmt2* mutant deficient in protein mannosylation had increased tolerance to AfpB, in correlation with the absence of protein interaction and internalization. Therefore, the mannoproteins surrounding the fungal cell could be mediating the interaction of AfpB with the cell wall, concentrating and bringing it near the plasma membrane for its internalization and cytotoxic activity. These results agree with previous observations with other antifungal peptides and proteins, such as synthetic PAF26 or plant osmotin, where cell wall mannoproteins were required for their activity against fungal cells (30, 32, 55). Moreover, our results support the idea that cell wall integrity is necessary for the activity of AfpB, as previously reported for the plant defensin NaD1 and osmotin (51, 56). Further study on the implication of cell wall mannoproteins in AfpB activity would help determine its spectrum of activity and the design of improved antifungal molecules.

AfpB and PAF interacted similarly and efficiently with the cell wall of *P. digitatum*, but AfpB is immediately internalized and exerts its cytotoxic effects inside the cell, whereas PAF remains attached to the extracellular envelope. These observations lead us to conclude that the capability of AfpB for rapid internalization is the determinant for its potent antifungal activity. We also show that AfpB internalization inside *P. digitatum* cells is an active process that requires energy, presumably via an endocytosis-like mechanism (Fig. 8). The active uptake of PAF and other antifungal peptides and proteins into susceptible cells has been previously observed (28, 29, 57). In the same way, the orthologous protein of AfpB in P. chrysogenum, PAFB, is actively taken up by the parental fungus, leading to autolysis, unlike PAF, to which the producer fungus is resistant (16). Therefore, the cell toxicity of this group of proteins correlates with active internalization in target fungi, and higher fungicidal activities are associated with proteins with efficient cell-penetrating capabilities. Future research to identify the determinants on the AFPs for efficient uptake into fungal cells will help to design potent antifungal proteins.

Our studies showed that AfpB causes a series of intracellular effects that lead to fungal cell death (Fig. 8). One of the first events was the generation of ROS, which was probably activated even earlier than AfpB cellular uptake, because ROS was quickly detected. ROS production occurred prior to cell death and not as a consequence of cell damage, suggesting that it has a signaling role. ROS accumulation seems to initiate signaling pathways that activated a transcriptional reprogramming to trigger a cell death program in the fungal cell. In addition to ROS, AfpB activates other signaling messengers, including mitogen-activated protein kinase cascades previously reported by our group (58), and G-protein signaling pathways, as indicated by the transcriptional activation of the α-subunit of the G-protein FadA detected in the present study. These results agree with a general role of G-protein signaling in the cytotoxicity of cell-penetrating AFPs, with previous research showing that *fadA* mutants from Aspergillus nidulans had reduced susceptibility to PAF (23) or NAFP (43) and reduced accumulation of G-proteins by PgAFP in Aspergillus flavus (52). This group of AFPs is reported to induce some morphological changes associated with programmed cell death, such as DNA fragmentation and phosphatidylserine externalization (23, 52). Similarly, AfpB-induced cell death was accompanied by a TUNEL-positive response, revealing DNA fragmentation associated with RCD. We went on to demonstrate that several regulated cell death markers are induced in response to AfpB in the fungal cells, including cell death execution methacaspases (*casA* and *casB*), proapoptotic genes (*nma*), and death inductive factors (*aif1* and *amid2*). We detected their transcriptional gene activation in response to AfpB in a reproducible quantitative manner, indicating that AfpB triggers an RCD program in its target fungi. Eventually, AfpB causes loss of plasma membrane integrity in fungal cells as a consequence of cell damage, but this is not its primary target. This information is of fundamental value in the biotechnological application of AfpB for guiding combination therapies and for evaluating secondary effects.

More intriguing is the biological function of AfpB in *P. digitatum*, a protein that is highly active against the parental fungus. AFPs have commonly been recognized as specific against fungi but innocuous or less active against the producer fungus (15). However, the discovery of AfpB (5) and, later, of the orthologous protein from P. chrysogenum PAFB (16) and AfpA from *P. expansum* (6), is bringing to light that self-activity of AFPs could be a more frequent phenomenon than thought. The finding that AfpB elicits a genetically encoded program of self-elimination makes us think that its biological role goes beyond being a weapon to fight competitors to being a weapon to ensure the survival of the whole species by regulating fungal populations. In this sense, based on a transcriptomic meta-analysis, it has been proposed recently that the AFPs from Aspergillus niger and A. giganteus are cannibal toxins in fungi with a primary function of killing genetically identical siblings for the benefit of the fungal community (22). A better understanding of the biological role of AFPs and how the producing organisms protect themselves from autolysis will broaden the knowledge of these valued antifungal molecules with relevant therapeutic applications.

## MATERIALS AND METHODS

### Strains, media, and culture conditions.

The wild-type *P. digitatum* PHI26 (CECT 20796) and the genetically engineered *Pdpmt2* disruptant (Δ*pmt2*) strains were used in this study (30, 59). They were cultured on potato dextrose agar (PDA) plates at 25°C for conidium collection. After dilution to the appropriate concentration, conidia were statically incubated in 1/10 potato dextrose broth (PDB) medium supplemented with 0.03% chloramphenicol during the indicated periods for antifungal assays and confocal microscopy studies.

### Protein production and purification.

Fully active AfpB and PAF proteins were produced in *P. digitatum* and purified to homogeneity as previously described (6, 19).

### Fluorescent labeling of AfpB and PAF.

Proteins were labeled via carboxyl groups with the fluorophore BODIPY-FL-EDA (Life Technologies), as previously described (51, 60). Unbound BODIPY and salts were removed through dialysis using benzoylated cellulose tubes (2,000 molecular weight cutoff; Sigma) against deionized water. Protein concentration was determined by UV spectrometry.

### Antifungal activity assays.

Growth inhibition assays were performed in 96-well microtiter plates as previously described (5). In these assays, antifungal proteins were normally incubated with conidia for 72 h to determine their growth-inhibitory activity. When indicated, the conidia were pregerminated for 24 h prior to incubation with the antifungal proteins. Fungicidal activity was assessed on conidial suspensions (10^4^ conidia/ml) in water by incubation with different protein concentrations per triplicate for 24 h at 25°C and low shaking. After treatment, samples were serially diluted and grown on PDA plates for 3 days to determine the number of viable conidia. Assays were repeated three times. IBM SPSS Statistics version 26 was used for statistical analyses, and one-way analysis of variance (ANOVA) and Tukey’s honestly significant difference (HSD) test were used to discriminate between treatments with 95% confidence.

### Confocal microscopy analysis.

The interaction of fluorescently labeled BODIPY-AfpB and BODIPY-PAF was analyzed on germlings originating from suspensions of 10^6^ conidia/ml germinated for 16 h, as described previously (28). To determine whether protein internalization is energy dependent, germinated conidia were pretreated with NaN_3_ (3 mM; Fluka) for 15 min. Fungal cell walls were stained with CFW (50 μg/ml; Fluka) for 15 min. ROS generation was visualized by incubating the AfpB- and PAF-treated fungal germlings with the ROS fluorescent probe CM-H_2_DCFDA (10 μM; Invitrogen). PI at 20 μg/ml (Sigma-Aldrich) and SYTOX green (5 μM) were used as dead cell staining. All these confocal studies were carried out in at least three independent assays and visualized in three independent samples each time (*n* = 9).

DNA fragmentation was evaluated using the TUNEL method as described previously, with minor modifications (40). Basically, the AfpB- or H_2_O_2_-treated hyphae were fixed in 4% paraformaldehyde directly on the treated media and bound to microscope slides coated with poly-l-lysine. Once dried, cell walls were digested at 37°C for 1 h with a lytic cocktail (16 mg/ml β-d-glucanase, 81.4 U/ml lyticase, 10 mg/driselase) and permeabilized for 15 min with 0.1% Triton X-100. The samples were then incubated with the solution provided by the *in situ* cell death detection kit, fluorescein (Roche). Finally, they were washed and stained with DAPI and observed under confocal microscopy.

Confocal laser scanning microscopy was with an Olympus FV1000 microscope (Olympus, Tokyo). BODIPY-AfpB and BODIPY-PAF, TUNEL stain, and ROS probe were excited at 488 nm with an argon ion laser, with the emission window set at 500 to 545 nm. The CFW and DAPI were excited at 405 nm and detected at 410 to 450 nm. The PI was excited with a laser diode at 559 nm and detected at 575 to 675 nm. Sequential bright-field images were captured with a transmitted light detector.

### ROS determination.

ROS production triggered by AfpB was monitored during a time lapse by measuring CM-H_2_DCFDA fluorescence using a SpectraMax M3 multimode microplate reader (Molecular Devices). Conidia (10^5^ in 100 μl 1/10 PDB) germinated for 16 h were incubated with 10 μM CM-H_2_DCFDA and AfpB at different concentrations, using water as a negative control or H_2_O_2_ as a positive control. Assays were carried out twice with 6 replicates per sample (*n* = 12).

### Gene expression analysis.

Total RNA was extracted from a 5-ml culture grown overnight, starting from 10^6^ conidia/ml, using the Maxwell RSC plant RNA kit (Promega). RNA (2 μg) was retrotranscribed using the high-capacity reverse transcription kit (Applied Biosystems). Quantitative reverse transcription PCR (qRT-PCR) analyses were carried out in 96-well optical plates in a LightCycler 480 System (Roche) using SYBR green and primers in Table S1 in the supplemental material. The results for gene expression were normalized to the β-tubulin gene values. Three technical replicates were done for each sample, and three independent assays were conducted. Statistical analysis was done using the R package (R-3.6.1) to compute the Student's *t* test at *P* values of ≤0.05 and 0.01.

10.1128/mSphere.00595-20.4 TABLE S1 Primers used for expression analysis of RCD-related genes. Download Table S1, DOCX file, 0.01 MB. Copyright © 2020 Bugeda et al. 2020 Bugeda et al. This content is distributed under the terms of the Creative Commons Attribution 4.0 International license.
