# Supplementary figures and images for: The Antifungal Protein AfpB Induces Regulated Cell Death in Its Parental Fungus Penicillium digitatum
Source: mSphere. 2020 Aug 26;5(4):e00595-20. doi: 10.1128/mSphere.00595-20 (PMC7449623; doi:10.1128/mSphere.00595-20)

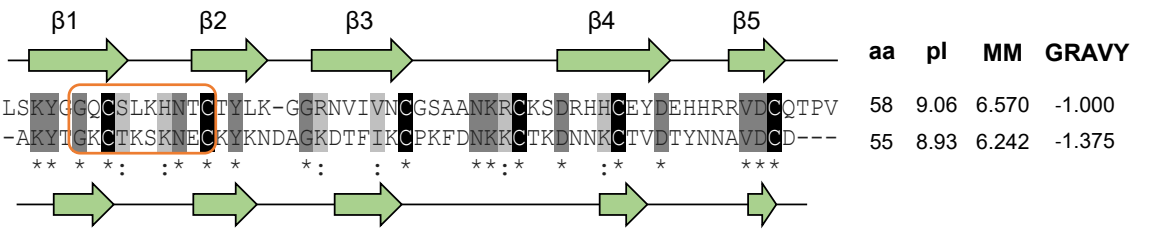

Supplement: FIG S1 [file mSphere.00595-20-sf001.pdf]

**A**

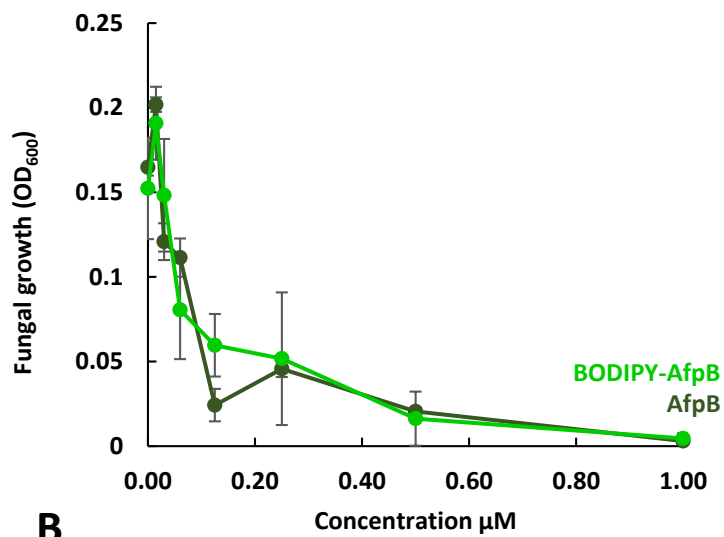

**B**

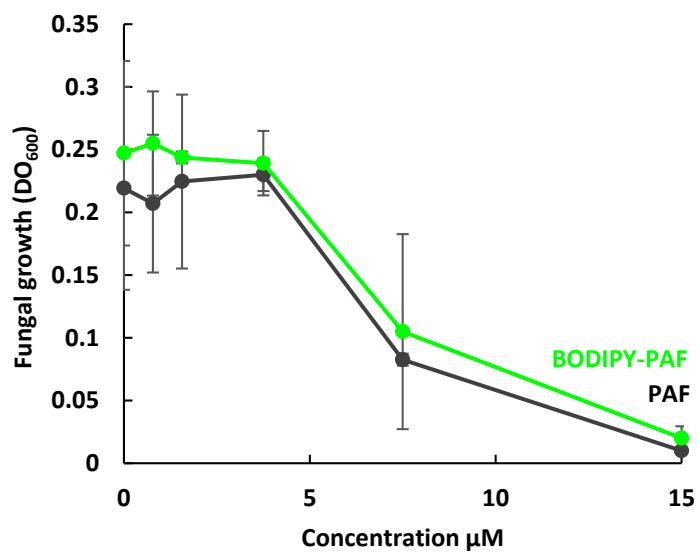

Supplement: FIG S2 [file mSphere.00595-20-sf002.pdf]

1.5  $\mu$ M AfpB  
+  
CM-H<sub>2</sub>DCFDA

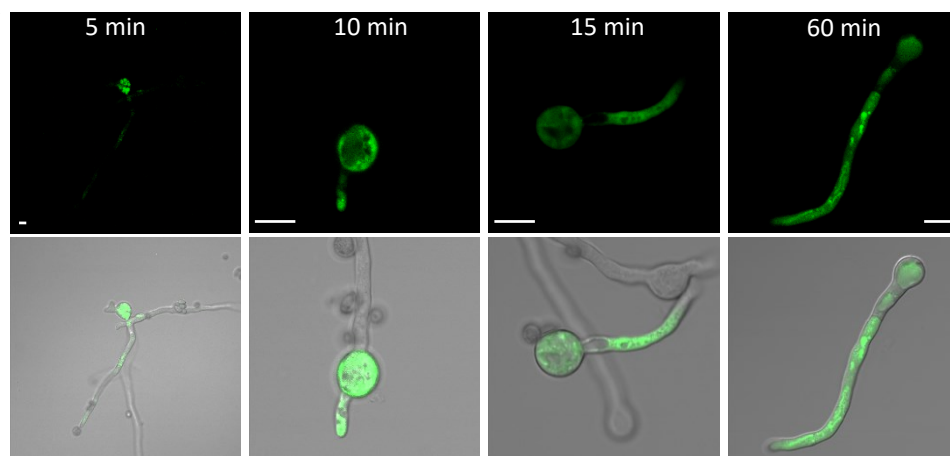

1.5  $\mu$ M AfpB  
+  
SYTOX green

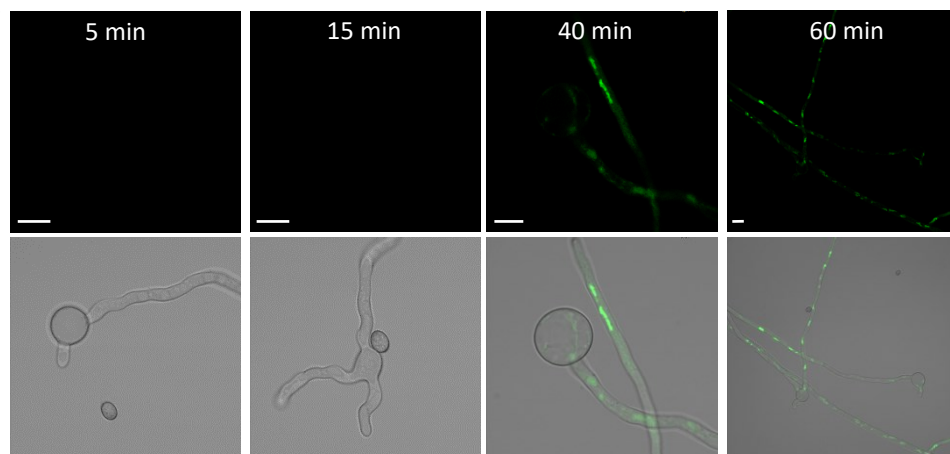

Supplement: FIG S3 [file mSphere.00595-20-sf003.pdf]
