# Supplementary material for: The Antifungal Protein AfpB Induces Regulated Cell Death in Its Parental Fungus Penicillium digitatum
Source: mSphere. 2020 Aug 26;5(4):e00595-20. doi: 10.1128/mSphere.00595-20 (PMC7449623; doi:10.1128/mSphere.00595-20)
Supplement: TABLE S1 [file mSphere.00595-20-st001.docx]

| Gene | Locus | Primers |
| --- | --- | --- |
|  |  |  |
| *β-tubulin* | PDIG_79010 | F: AGCGGTGACAAGTACGTTCC |
|  |  | R: CTTGCCAAAGGGACCGGAG |
| *casA* | PDIG_60552 | F: GGCCTACTTGGTGTGGTTT |
|  |  | R: CATACACTTCGTCGCCTTTGG |
| *casB* | PDIG_22040 | F: CAGCAATTCGGTGGAGGGG |
|  |  | R: CGGCTGTCCTGCATAGTTGA |
| *fadA* | PDIG_78030 | F: ACGATCCTGAAGCAGATGAAG |
|  |  | R: TAACCCGCATCGACTGGAC |
| *nma* | PDIG_75700 | F: GTCGTAGCCAAAACTACCGTC |
|  |  | R: GCGTCGAGGTTGATTGCTC |
| *aif1* | PDIG_18400 | F: CCAGTCAACGGGTATGATGATGT |
|  |  | R: CCATAGTAGCAACCGCCACA |
| *amid-2* | PDIG_76640 | F: ACTCAGCCCTCCATGACCTT |
|  |  | R: GAATCCACTCGGCGGAACAA |

**Table S1.** Primers used for expression analysis of RCD-related genes.
